# Do women living in a deprived neighborhood have higher maternity care costs and worse pregnancy outcomes? A retrospective population-based study

**DOI:** 10.1186/s12913-024-10737-2

**Published:** 2024-03-20

**Authors:** Eline K. Nanninga, Malou D. Menting, Eric J. E. van der Hijden, France R. M. Portrait

**Affiliations:** 1 School of Business and Economics Department of Health Sciences, Ethics, Governance and Society, Vrije Universiteit Amsterdam, De Boelelaan 1085, 1081 HV Amsterdam, The Netherlands; 2 Dutch Healthcare Authority, Newtonlaan 1-41, 3584 BX Utrecht, The Netherlands; 3 Zilveren Kruis Health Insurance , Dellaertweg 1, 2316 WZ Leiden, The Netherlands

**Keywords:** Maternity care, Perinatal costs, Deprived neighborhoods, Pregnancy

## Abstract

**Background:**

Living in a deprived neighborhood is associated with poorer health, due to factors such as lower socio-economic status and an adverse lifestyle. There is little insight into whether living in deprived neighborhood is associated with adverse maternity care outcomes and maternity health care costs. We expect women in a deprived neighborhood to experience a more complicated pregnancy, with more secondary obstetric care (as opposed to primary midwifery care) and higher maternity care costs. This study aimed to answer the following research question: to what extent are moment of referral from primary to secondary care, mode of delivery, (extreme or very) preterm delivery and maternity care costs associated with neighborhood deprivation?

**Methods:**

This retrospective cohort study used a national Dutch database with healthcare claims processed by health insurers. All pregnancies that started in 2018 were included. The moment of referral from primary to secondary care, mode of delivery, (extreme or very) preterm delivery and maternity care costs were compared between women in deprived and non-deprived neighborhoods. We reported descriptive statistics, and results of ordinal logistic, multinomial and linear regressions to assess whether differences between the two groups exist.

**Results:**

Women in deprived neighborhoods had higher odds of being referred from primary to secondary care during pregnancy (adjusted OR 1.49, 95%CI 1.41–1.57) and to start their pregnancy in secondary care (adjusted OR 1.55, 95%CI 1.44–1.66). Furthermore, women in deprived neighborhoods had lower odds of assisted delivery than women in non-deprived neighborhoods (adjusted OR 0.73, 95%CI 0.66–0.80), and they had higher odds of a cesarean section (adjusted OR 1.19, 95%CI 1.13–1.25). On average, women in a deprived neighborhood had higher maternity care costs worth 156 euros (95%CI 104–208).

**Conclusion:**

This study showed that living in a deprived neighborhood is associated with more intensive maternal care and higher maternal care costs in the Netherlands. These findings support the needs for greater attention to socio-economic factors in maternity care in the Netherlands.

**Supplementary Information:**

The online version contains supplementary material available at 10.1186/s12913-024-10737-2.

## Introduction

Maternity care in the Netherlands has been under close scrutiny since 2004. At that time, the country scored low in the European ranking with relatively high fetal mortality rates^1^ of 7.0 per 1000 births [1]. In subsequent European comparisons, the country scored better, with its lowest fetal mortality rate of 4.4 per 1000 livebirths in 2016 [2]. Since 2016, however, the rate has again been increasing. In 2021, it was back at 5.0 fetal deaths per 1000 livebirths [2]. In the most recent European ranking, the Netherlands ranks in the middle of the range [3]. Socio-economic factors have been shown to be important determinants of pregnancy outcomes and maternity care costs [4]. Therefore, it is crucial to further investigate the role of these factors in maternity care to improve its quality [4]. In this study, maternity care refers to care during pregnancy, care during delivery and care up to six weeks after delivery for both the mother and child.

Socioeconomic status (SES) can be defined as a combination of “the conditions in which people are born, grow up, live, work and age” [5]. Factors that are usually taken into account to assess SES are income, (un)employment, education level, race or migration status [6]. Women with lower SES use on average less prenatal care than others [7]. They also have adverse pregnancy and delivery outcomes more often, such as pregnancy induced hypertension, cesarean section, preterm delivery, babies with low birth weight, babies with a low Apgar score^2^, or perinatal death [7–12]. These unfavorable pregnancy outcomes may result in serious health consequences for the child later in life, stressing the need for more attention towards supporting pregnant women with lower SES (“vulnerable women”) [13, 14]. 

SES disadvantaged groups tend to cluster in deprived neighborhoods [15]. Furthermore, living in a deprived neighborhood can also have adverse effects on people’s health, besides one’s individual SES and lifestyle. Air pollution, poor housing, and community effects of a deprived neighborhood, can add on to the health risks people face [16–19]. 

If living in disadvantaged neighborhoods is also positively associated with adverse pregnancy and infant outcomes, neighborhood characteristics may be taken into account when designing public health interventions to improve maternal and child health [12]. Empirical literature has shown that women living in a disadvantaged neighborhood may be more likely to have adverse pregnancy and birth outcomes than others [20–30]. Most importantly, they have on average higher rates of perinatal mortality. Results of studies are inconclusive about the link between pregnancy induced hypertension and women living in deprived neighborhoods [20, 21, 23–25, 28, 29]. Additionally, the children of women living in deprived neighborhoods have on average lower Apgar scores, are more often born premature (< 37 weeks) and are more often small for gestational age [20–22, 27–29]. The link between neighborhood deprivation and a selection of adverse pregnancy and birth outcomes has also been established in the Netherlands [20, 24–26, 29]. 

However, to our knowledge, there is still no or little insight into whether other important maternity care outcomes (such as extreme or very preterm birth, cesarean section, prenatal care use) and maternity care costs are linked to neighborhood deprivation. As the Dutch maternity care system is unique in the distinction between care providers for low risk (primary care) and high risk (secondary care) pregnancies, there is little research on the difference in the moment of referral to high-risk care between women living in deprived and non-deprived neighborhoods [31]. Furthermore, previous research is inconclusive about the risk of cesarean section for women with a lower versus higher income, with no research about the risk of assisted deliveries [8, 30]. Finally, little is known about the odds of preterm delivery before 32 weeks or even 26 weeks gestation [1–3, 6]. This study aims to assess the relationships between neighborhood deprivation and maternity care outcomes, in light of the recent increase in adverse outcomes in the Netherlands [2]. More specifically, we focus on outcomes that have not yet been studied in detail: extreme or very preterm delivery, cesarean section rates, and partly related to the previous outcomes: maternity care costs per patient (namely care during pregnancy and delivery and care up to six weeks after delivery) and the moment of referral to secondary care. We expect women in deprived neighborhoods to experience a more complicated pregnancy, with more secondary obstetric care (as opposed to primary midwifery care) and higher maternity care costs. Therefore, the main research question of the current paper is: to what extent are moment of referral from primary to secondary care, mode of delivery, (extreme or very) preterm delivery and maternity care costs associated with neighborhood deprivation?

## Contextual background

Currently, the Dutch maternity care system is built around primary care midwives as main maternity care providers, who refer to obstetricians and clinical midwives (secondary care) when pregnancy or delivery becomes high-risk or when complications arise. Women with low-risk pregnancies can deliver at home, in a birthing center or at the hospital. In all cases of low-risk deliveries, the primary care midwife provides assistance, regardless of the location of delivery. After referral to secondary care, the primary care midwife is no longer responsible for the patient. Typically, the patient’s care is transferred back to the primary care midwife after the delivery. During the delivery and the first eight to ten days after delivery, a maternity nurse assists the new family at home (this assistance is called postnatal maternity help) [32]. The different healthcare providers are each funded through different cost systems. Primary midwifery care is predominantly paid using a bundle payment for the full trajectory and sub-trajectories of maternity care, except for a few specific activities, such as ultrasounds. The Dutch Healthcare Authority (“Nederlandse Zorgautoriteit”, NZa) sets yearly a nationwide maximum price for the bundles [33]. Secondary care provided by obstetricians and clinical midwives is financed using the Diagnosis Treatment Combinations (“DBC” in Dutch) system, a variant on the Diagnosis Related Groups (DRG) system used internationally [34]. The price of a DBC is based on all care activities that are on average performed per diagnosis. DBC prices are negotiated yearly between healthcare providers and private health insurers [35]. Postnatal maternity help is predominantly paid at an hourly rate, with the nationwide maximum price also determined by the NZa [36]. In 2015, integrated maternity care organizations (IGOs) were introduced. Maternity care providers in primary care, secondary care, and postnatal maternity help are all part of the IGO. These health care providers collaborate to offer comprehensive care from pregnancy until six weeks post-delivery. Instead of using separate cost systems (mono-disciplinary payment system), these care providers are paid by the IGO which in turn is funded by the health care insurer. The IGO receives a bundled payment for the IGO per woman from the health care insurer (integrated payment system) [37]. As the vast majority of maternity care is still provided using the mono-disciplinary system, this article discusses the findings within this context.

The Dutch government and the NZa aim to compensate healthcare providers who treat disproportionally more patients with lower SES than others, as these providers spend on average more time and resources on these patients than on other patients [38]. Qualification for the compensation from the Dutch government is currently based on the rate of neighborhood deprivation at the place of residence of the individual [33, 38]. Primary care midwives receive a 23% surcharge per woman living in deprived neighborhoods [33]. During the yearly negotiation between hospitals performing secondary care and health insurers, special agreements can be made for the price charged for care for people from deprived neighborhoods. A compensation is often also negotiated between health insurers and postnatal maternity help organizations for the postnatal maternity help fees, in the form of a 10% surcharge [36]. 

## Methods

### Datasets and study design

This retrospective cohort study used data from Vektis, data from “DBC-information system”-data (DIS), and data from Statistics Netherlands. Vektis, the executive agency of the Dutch healthcare insurers, receives all (primary, secondary and tertiary care) claims processed by all health insurers [39]. We had access to all claims with obstetric DBC codes, and for midwifery and postnatal maternity help in the period ranging from January 1, 2015 to December 31, 2021. As health insurance for a basic healthcare package is mandatory, the Vektis dataset covered almost the entire Dutch population (99.8%) [40, 41]. The claim data also provided limited individual demographic information (i.e., year of birth and 4-digit postal code) and the sum of total healthcare costs per year. The DIS data was available from January 1, 2018 to December 31, 2019. The DIS data provided additional information at an individual level on the diagnoses of the secondary care codes from Vektis. Public data from Statistics Netherlands was used to account for the degree of urbanization of the women’s neighborhood.

All data was at the individual level and was pseudonymized, preventing it from being traceable to an individual. Pseudonymization was performed using personal identification numbers, which made it possible to merge the claim data and DIS data at the individual level.

### Study sample

This study investigated pregnancy trajectories from conception until the first two months after delivery. We selected all pregnancies that started in the year 2018. More recent data was likely biased by changes in access to care in 2020 due to the COVID-19 pandemic, and/or was still incomplete at the start of this study due to administration delays.

Women were included when a delivery was registered in the claim data, either by the primary care practice or by the secondary care unit. Subtracting 9 months (or 7 or 6 months when preterm delivery was registered) from the date of delivery gave an approximate start date for the pregnancy. Pregnancy trajectories that ended in delivery (> 16 weeks) in the Netherlands were registered. Miscarriages, medical abortions, and pregnancies where delivery was outside of the Netherlands were not included.

### Variables

#### Dependent variables: pregnancy outcome and costs of pregnancy care

Maternal care outcomes were measured using four variables: moment of referral from primary to secondary care, mode of delivery, (extreme or very) preterm delivery, and maternity care costs.

The moment of referral from primary to secondary care was categorized into four categories: no referral (indicating having received care from a primary care midwife and no secondary care), referral during delivery or postpartum, referral during pregnancy, and start in secondary care (no primary care midwife was involved). Formally, in the current Dutch healthcare system, the primary care midwife no longer bears responsibility for the client after referral, and they cannot charge the health insurer for any provided care at this point. However, in practice, shared care is quite common. The mode of delivery was categorized in three categories (natural delivery, assisted delivery or cesarean section– ordinal variable). Preterm delivery was also categorized in three categories ((close to) term > 32 weeks gestation, very preterm < 32 weeks gestation or extremely preterm < 26 weeks gestation– ordinal variable). International studies show a higher risk of delivery before 37 weeks gestation for women in deprived neighborhoods [20–29]. This study specifically addresses the less-explored domain of (extreme) prematurity (< 32 weeks). The DBC-codes only allowed to identify three different periods of delivery (< 26 weeks, 26–32 weeks and > 32 weeks). It was however not possible to distinguish between deliveries at 32–37 weeks (mild prematurity) and those after 37 weeks of gestation. Total costs per pregnancy trajectory were computed by summing up all primary care, secondary care and postnatal maternity help invoices between 9 months (or 6 or 7 months for preterm delivery) before and two months after delivery. Costs were also computed per provider category (i.e., primary care, secondary care, postnatal maternity help, or integrated maternity care organizations) and for the whole trajectory. The price for primary care was slightly corrected. The applied rates in 2018 were deemed too high by the NZa [42]. Therefore, a nationwide one-time reduction of the rates was performed in 2019 as compensation [42]. We retrospectively corrected the 2018 rates to the intended rate and removed the one-time reduction from the 2019 rate.

#### Main independent variable

This study compared women living in deprived neighborhoods with women living in non-deprived neighborhoods. Classification was based on the classification system from the Dutch Healthcare Authority (NZa). The NZa labels neighborhoods into deprived and non-deprived neighborhoods, based on the percentage of inhabitants with a low income, the percentage of inhabitants who are unemployed, and the percentage of inhabitants with a non-Western migration background [38, 43]. Each neighborhood receives a deprivation-score, based on these factors. This classification is re-evaluated yearly. Each year the cut-off for neighborhood deprivation is at the inclusion of 1.6 million inhabitants in deprived neighborhoods [43]. Classification of living in a deprived neighborhood was registered in our dataset per quarter in 2018 and 2019. Please note that the classification from 2018 was based on more and other variables than the ones used in 2019, and that these are not used anymore [44]. Because of that, we used the deprivation classification of 2019 for all women included in our sample: only women registered as living in a deprived neighborhood in all four quarters in 2019 were classified as living in a deprived neighborhood during her pregnancy.

#### Other independent variables

We controlled for the following four variables available in our dataset, based on previous research on the factors that are correlated with maternity care outcomes and healthcare costs. First, we included the woman’s age at the estimated start of pregnancy. Maternal age is an indicator of healthcare use and costs, and women with lower SES often get pregnant at younger ages than women with higher SES [45, 46]. Maternal age was characterized using four categories: (1) younger than 25 years, (2) between 25 and 30, (3) between 30 and 35, and (4) 35 years or older. Second, we used the variable 2017 healthcare costs. This was used as a proxy for health status at the beginning of the pregnancy. The costs were divided into six equally sized groups, and five dummies were included in the analyses (reference category was 2017 healthcare costs below €123). Previous health issues could lead to worse pregnancy outcomes [47, 48]. Third, we included the population density of the women’s neighborhood. Population density is linked to accessibility to maternal health services, with less access in low density areas [49]. We used the 4-digit postal code of the women to determine the population density, based on classification by Statistics Netherlands. Population density was categorized using five dummies: (1) Very strongly urbanized (≥ 2500 addresses/km^2^), (2) strongly urbanized (1500–2500 addresses/km^2^), (3) moderately urbanized (1000–1500 addresses/km^2^), (4) hardly urbanized (500–1000 addresses/km^2^), and (5) not urbanized (< 500 addresses/km^2^). Moderately urbanized was used as the reference category. Fourth, we included the year of delivery to adjust for fee differences between 2018 and 2019 due to inflation, indexation, and new negotiated prices between care providers and health insurers. In the present study, we did not have access to other relevant demographic and health characteristics of the women as factors such as parity, lifestyle, and medical or family history were not available.

### Statistical analyses

Descriptive analyses for moment of referral, mode of delivery, (extreme or very) preterm delivery, maternity care costs, maternal age, healthcare costs in 2017, and population density were performed. The mean and standard deviation (SD) or median and inter-quartile range (IQR) for continuous and count variables, and percentages for categorical variables were computed. Differences between the deprived and non-deprived neighborhoods were assessed using two sample t-tests, Mann-Whitney U tests and chi-square tests where appropriate. Second, we estimated four different models (i.e., moment of referral, mode of delivery, preterm delivery, and maternity care costs). For continuous outcome variables, we used multivariable linear regression, and for ordinal variables, we used ordinal logistic regression. For the ordinal logistic regressions, we checked the proportional odds assumption. When the p-value was below 0.05, we ran a multinomial logistic regression. For all these models, crude models were estimated as well as models adjusted for the case-mix factors. Finally, in the sensitivity analyses, all statistical analyses were repeated with a slightly different classification for deprived neighborhood. In these analyses, living in a deprived area in 2019 for one or more quarters was considered sufficient to be classified as living in a deprived neighborhood (sensitivity analysis). Statistical significance was set at p-value < 0.05. Data cleaning, manipulation, and statistical analyses were performed using R 4.1.2 with RStudio [50]. 

### Ethical approval

Ethical approval from the Medical Ethics Assessment Committee (METC) was not required for this study. We used existing, administrative claims data for this study, and thus conducted no clinical trials or other activities with human participants. The NZa is approved to gather cost data and to execute market research when this falls within the legal task performance of the organization.

## Results

### Descriptive statistics

Table 1 reports descriptive statistics for all included women and stratified by type of neighborhood. 163,136 women with an estimated pregnancy start in 2018 were included. Their mean age was 30.7 years old (SD 4.7) at the start of the pregnancy. 12,946 women (7.9% of included population) lived in a deprived neighborhood for all four quarters of 2019. The average age was lower for women in deprived neighborhoods, and the median for healthcare costs in 2017 was higher. Finally, the population density in deprived neighborhoods was higher than for non-deprived neighborhoods, with 49.6% of women in deprived neighborhoods in very strongly urbanized areas, compared to 20.1% in non-deprived neighborhoods.

The moment of referral in women in deprived neighborhoods was significantly earlier than for women in non-deprived neighborhoods; more women started in secondary care (13.1% vs. 10.8%), and more were referred during pregnancy (41.2% vs. 34.5%). There were relatively more women in non-deprived neighborhoods who received only primary care (25.4% vs. 19.6%) (Table 1). The mode of delivery for the majority of women was natural delivery (78.3%). Women in deprived neighborhoods had a cesarean section more often (18.1% vs. 15.5%), and women in non-deprived neighborhoods had relatively more assisted deliveries (6.1% vs. 4.7%) (Table 1). There was no significant difference in the number of extreme or very preterm deliveries (< 32 weeks) between the types of neighborhood. The median of total maternity care costs was higher for women in deprived neighborhoods than for women in non-deprived neighborhoods. Division of the costs over the different care domains shows that the maternity care costs in deprived neighborhoods were higher for secondary care, and lower for both primary care and postnatal maternity help.

**Table 1:** Descriptive statistics of total population and stratified by type of neighborhood

	Total population	Deprived neighborhood	Non-deprived neighborhood	P-value^†^
	*N* = 163,136	*N* = 12,946	*N* = 150,190	
**Outcome variables**				
Moment of referral (%)				< 0.001*
No referral	25.0	19.6	25.4	
During delivery or postpartum	28.7	25.7	29.0	
During pregnancy	35.0	41.2	34.5	
Start in secondary care	11.0	13.1	10.8	
Missing	0.3	0.4	0.3	
Mode of delivery (%)				< 0.001*
Natural delivery	78.3	77.2	78.4	
Assisted delivery	6.0	4.7	6.1	
Cesarean section	15.7	18.1	15.5	
Preterm delivery (%)				0.11
(Close to) term > 32 weeks	99.5	99.4	99.5	
Very preterm 26–32 weeks	0.4	0.4	0.4	
Extreme preterm < 26 weeks	0.1	0.2	0.1	
Maternity care costs (median in € (IQR))				
Primary care	1624 (1187–1786)	1511 (1032–1886)	1625 (1195–1784)	< 0.001*
Secondary care	3162 (2248–4895)	3530 (2391–5208)	3135 (2237–4863)	< 0.001*
Integrated maternity care organizations	4945 (3887–6481)	4945 (3924–6316)	4938 (3882–6485)	0.56
Postnatal maternity help	2202 (1606–2375)	1731 (1138–2375)	2216 (1672–2544)	< 0.001*
Total maternity care	6617 (5304–7946)	6700 (5459–8137)	6612 (5287–7929)	< 0.001*
**Case-mix variables**				
Age				< 0.001*
Mean in years (sd)	30.7 (4.7)	30.1 (5.4)	30.7 (4.6)	
Healthcare costs 2017				< 0.001*
Median in € (IQR)	612 (172–2999)	773 (229–3196)	599 (168–2981)	
Population density (%)				< 0.001*
Very strongly urbanized	22.4	49.6	20.1	
Strongly urbanized	21.0	25.9	20.6	
Moderate urbanized	15.6	7.8	16.2	
Hardly urbanized	14.8	2.3	15.9	
Not urbanized	12.0	0.5	12.9	
Missing	14.2	13.9	14.9	

^†^P-values of calculated differences in type of neighborhood with two sample t-tests or Mann-Whitney U, and chi-square tests on differences in type of neighborhood

*P-value lower than alpha of 0.05

### Statistical analyses

#### Moment of referral

In our analysis of the moment of referral to secondary care, the ordinal logistic regression did not meet the proportional odds assumption. Therefore, Table 2 shows the estimation results of a multinomial regression model for moment of referral, with no referral in pregnancy as reference category. Women in deprived neighborhoods had higher odds to be referred to secondary care during pregnancy (adjusted OR 1.49, 95%CI 1.41–1.57) and higher odds to start their pregnancy in secondary care (adjusted OR 1.55, 95%CI 1.44–1.66). There was no significant difference in referral during delivery or postpartum, compared to no referral, between the women in deprived and non-deprived neighborhoods. Higher age seemed to decrease the odds for referral during delivery, or during pregnancy, and increase the odds of starting in secondary care when the woman was 35 years or older. Living in an urbanized area increased the odds of referral during delivery, and women in less urbanized neighborhoods had lower odds of referral during delivery.

**Table 2:** Model 1 Multinomial regression moment of referral, no referral as reference category

	Referral at delivery or postpartum				Referral during pregnancy				Start in secondary care			
	Crude model		Adjusted model		Crude model		Adjusted model		Crude model		Adjusted model	
	OR (95%CI)	P-value	OR (95%CI)	P-value	OR (95%CI)		OR (95%CI)	P-value			OR (95%CI)	P-value
Deprived neighborhood	1.15 (1.09–1.21)	< 0.001*	1.00 (0.94–1.07)	0.92	1.54 (1.47–1.62)	< 0.001*	1.49 (1.41–1.57)	< 0.001*	1.58 (1.48–1.68)	< 0.001*	1.55 (1.44–1.66)	< 0.001*
**Age**												
Younger than 25 years			Ref				Ref				Ref	
25–30 years			0.78 (0.73–0.82)	< 0.001*			0.84 (0.79–0.88)	< 0.001*			0.98 (0.90–1.07)	0.67
30–35 years			0.60 (0.57–0.64)	< 0.001*			0.77 (0.73–0.81)	< 0.001*			0.98 (0.90–1.06)	0.64
35 years or older			0.54 (0.51–1.57)	< 0.001*			0.98 (0.92–1.04)	0.43			1.56 (1.43–1.70)	< 0.001*
**Healthcare costs 2017**												
< €123			Ref				Ref				Ref	
€123– €254			1.04 (0.99–1.09)	0.09			1.14 (1.09–1.20)	< 0.001*			0.94 (0.86–1.01)	0.10
€254– €612			1.20 (1.14–1.26)	< 0.001*			1.45 (1.38–1.52)	< 0.001*			1.43 (1.32–1.54)	< 0.001*
€612– €1742			1.23 (1.17–1.29)	< 0.001*			1.67 (1.59–1.75)	< 0.001*			2.27 (2.11–2.44)	< 0.001*
€1742– €4911			0.72 (0.69–0.76)	< 0.001*			1.05 (0.99– 1.10)	0.06			1.74 (1.63–1.87)	< 0.001*
> €4911			0.80 (0.76–0.84)	< 0.001*			1.79 (1.71–1.88)	< 0.001*			3.32 (3.10–3.56)	< 0.001*
**Population density**												
Very strongly urbanized			1.16 (1.11–1.21)	< 0.001*			0.96 (0.91–0.99)	0.02*			0.80 (0.75–8.85)	< 0.001*
Strongly urbanized			1.08 (1.03–1.13)	< 0.01*			1.04 (0.99–1.08)	0.07			0.99 (0.94–1.06)	0.87
Moderately urbanized			Ref				Ref				Ref	
Hardly urbanized			0.95 (0.90–0.99)	0.03*			0.96 (0.92–1.01)	0.90			0.80 (0.75–0.85)	< 0.001*
Not urbanized			0.84 (0.79–0.89)	< 0.001*			0.93 (0.89–0.98)	< 0.01*			0.78 (0.73–0.84)	< 0.001*

*P-value lower than alpha of 0.05

#### Mode of delivery

Table 3 shows the estimation results of the model for the differences in mode of delivery between women in deprived and in non-deprived neighborhoods. The ordinal logistic regression did not meet the proportional odds assumption (p-value < 0.05). Therefore, we used a multinomial logistic regression, with natural delivery as reference category. Table 3 shows that women in deprived neighborhoods had lower odds of assisted delivery than women in non-deprived neighborhoods (adjusted OR 0.73, 95%CI 0.66–0.80), and they had higher odds of a cesarean section (adjusted OR 1.19, 95%CI 1.13–1.25). Both parameters were statistically significant.

**Table 3:** Model 2 Multinomial regression mode of delivery, adjusted for case-mix factors

	Assisted delivery				Cesarean section			
	Crude model		Adjusted model		Crude model		Adjusted model	
	OR (95%CI)	P-value	OR (95%CI)	P-value	OR (95%CI)	P-value	OR (95%CI)	P-value
Deprived neighborhood	0.79 (0.72–0.86)	< 0.001*	0.73 (0.66–0.80)	< 0.001*	1.19 (1.13–1.24)	< 0.001*	1.19 (1.13–1.25)	< 0.001*
**Age**								
Younger than 25 years			Ref				Ref	
25–30 years			1.12 (1.03–1.22)	< 0.01*			1.27 (1.19–1.36)	< 0.001*
30–35 years			0.92 (0.85–1.00)	0.05			1.49 (1.40–1.59)	< 0.001*
35 years or older			0.78 (0.71–0.86)	< 0.001*			2.04 (1.91–2.18)	< 0.001*
**Healthcare costs 2017**								
< €123			Ref				Ref	
€123– €254			0.93 (0.86–0.99)	0.04			1.13 (1.07–1.19)	< 0.001*
€254– €612			0.98 (0.91–1.05)	0.52			1.24 (1.18–1.31)	< 0.001*
€612– €1742			0.95 (0.89–1.03)	0.20			1.30 (1.23–1.37)	< 0.001*
€1742– €4911			0.65 (0.60–0.70)	< 0.001*			1.04 (0.98–1.10)	0.16
> €4911			0.56 (0.51–0.61)	< 0.001*			1.43 (1.35–1.50)	< 0.001*
**Population density**								
Very strongly urbanized			1.22 (1.14–1.31)	< 0.001*			1.05 (1.00–1.10)	0.04
Strongly urbanized			1.13 (1.06–1.22)	< 0.001*			1.07 (1.02–1.12)	< 0.01*
Moderately urbanized			Ref				Ref	
Hardly urbanized			1.00 (0.93–1.08)	0.94			1.00 (0.95–1.05)	0.94
Not urbanized			1.00 (0.92–1.08)	0.98			1.02 (0.97–1.08)	0.49

*P-value lower than alpha of 0.05

#### (Extreme/very) preterm delivery

Table 4 reports the estimation results for the pregnancy outcome preterm delivery. The proportional odds assumption was met (p-value ≥ 0.05). The ordinal logistic regression showed that there was no statistically significant difference between the deprived and non-deprived neighborhoods in the odds of very preterm delivery and extremely preterm delivery, compared to delivery after 32 weeks gestation (p-value = 0.19). However, note that the coefficients are larger than one, which suggests higher rates of extreme or very preterm delivery in deprived neighborhoods.

**Table 4:** Model 3 Ordinal regression preterm delivery

	Crude model OR (95%CI)	P-value	Adjusted model OR (95%CI)	P-value
Deprived neighborhood	1.18 (0.92–1.48)	0.18	1.19 (0.91–1.55)	0.19
**Age**				
Younger than 25 years			Ref	
25–30 years			0.94 (0.71–1.27)	0.69
30–35 years			0.94 (0.72–1.26)	0.68
35 years or older			0.94 (0.70–1.29)	0.72
**Healthcare costs 2017**				
< €123			Ref	
€123– €254			1.20 (0.90–1.63)	0.23
€254– €612			1.50 (1.13–2.01)	0.006*
€612– €1742			1.47 (1.11–1.97)	0.009*
€1742– €4911			1.50 (1.13–2.00)	0.006*
> €4911			1.89 (1.44–2.51)	< 0.001*
**Population density**				
Very strongly urbanized			1.27 (1.00–1.62)	0.05
Strongly urbanized			1.11 (0.87–1.42)	0.41
Moderately urbanized			Ref	
Hardly urbanized			1.03 (0.78–1.35)	0.82
Not urbanized			1.30 (0.99–1.71)	0.05

Preterm delivery was classified in three categories: (close to) term delivery (> 32 weeks, reference category), very preterm delivery (26–32 weeks gestation), extreme preterm delivery (< 26 weeks gestation).* P-value lower than alpha of 0.05

#### Maternity care costs

Table 5 presents the estimation results of the linear model with the total maternity care costs per pregnancy as a dependent variable and neighborhood classification as independent variable, with correction for the contextual changes over the years, by adding year of the delivery to the model. The crude model shows that there was a statistically significant difference in maternity care costs. Women living in a deprived neighborhood use on average 204 euros more maternity care (95%CI 157–250) than women in non-deprived neighborhoods. The model adjusted for case-mix factors shows that, on average, women in a deprived neighborhood use 156 euros more maternity care (95%CI 104–208). The case-mix factor healthcare costs 2017 shows that women in groups with higher previous healthcare costs had higher maternity care costs.

**Table 5:** Model 4 Linear regression total maternity care costs, in euros

	Crude model		Adjusted model	
	Coefficients (95%CI)	P-value	Coefficients (95%CI)	P-value
Deprived neighborhood	204 (157–250)	< 0.001*	156 (104–208)	< 0.001*
Year of delivery 2019	152 (123–181)	< 0.001*	146 (116–176)	< 0.001*
**Age**				
Younger than 25 years			ref	
25–30 years			24 (−29 to 77)	0.36
30–35 years			6 (−45 to 58)	0.81
35 years or older			231 (175–286)	< 0.001*
**Healthcare costs 2017**				
< €123			Ref	
€123–€254			174 (127–221)	< 0.001*
€254–€612			378 (331–425)	< 0.001*
€612–€1742			574 (527–621)	< 0.001*
€1742–€4911			311 (264–358)	< 0.001*
> €4911			732 (685–780)	< 0.001*
**Population density**				
Very strongly urbanized			−23 (−65 to 19)	0.28
Strongly urbanized			13 (−29 to 55)	0.54
Moderately urbanized			Ref	
Hardly urbanized			−8 (−54 to 37)	0.72
Not urbanized			−18 (−66 to 30)	0.46
Intercept	6797 (6772–6823)	< 0.001*	6412 (6345–6479)	< 0.001*

*P-value lower than alpha of 0.05

### Sensitivity analysis

For the sensitivity analysis, we considered women who had lived in a deprived neighborhood for at least one quarter of 2019 as living in a deprived neighborhood during their full pregnancy. This changed the distribution of women living in a (non-)deprived neighborhood (10.6% in deprived neighborhoods and 89.4% in non-deprived neighborhoods). The sensitivity analysis showed a stronger association in the same direction between neighborhood deprivation and the outcomes mode of delivery, preterm delivery, and maternity care costs. The coefficient of the moment of referral became statistically significant for referral during delivery or postpartum: women living in a deprived neighborhood were referred to secondary care more often (OR 1.07, 95%CI 1.01–1.12). Women living in a deprived neighborhood had significantly more very preterm (< 32 weeks) and extreme preterm (< 26 weeks) deliveries (OR 1.30, 95%CI 1.02–1.64). Tables of the sensitivity analysis are shown in the supplementary information.

## Discussion

This study assessed the association between neighborhood deprivation and three maternity care outcomes and maternity care costs in the Netherlands. We observed that women living in deprived neighborhoods were more often referred to secondary care at the start of and during their pregnancy and more often received a cesarean section. Furthermore, the costs of maternity care were higher for women living in deprived neighborhoods than for women living in non-deprived neighborhoods. Neighborhood deprivation was not associated with delivering very or extreme preterm < 32 weeks (except in the sensitivity analyses, where we used a less stringent definition of living in a deprived neighborhood).

Our finding that women in deprived neighborhoods were treated in secondary care more often suggests that these women required extra monitoring during pregnancy, either due to previous health issues or complications in previous pregnancies, or due to (risk of) complications in the current pregnancy. This is consistent with existing literature that indicates that women living in deprived neighborhoods have higher risks of complications in pregnancy and delivery [20–30]. In addition, Klumper and colleagues found that women in deprived neighborhoods more often are multiparous [26]. As outcomes of previous pregnancies can lead to indications to start a following pregnancy in secondary care, this too may partially explain the observed higher odds of starting pregnancy in secondary care in deprived neighborhoods.

The observed higher rate of cesarean sections for women living in a deprived neighborhood agrees with literature on women with lower SES, showing that lower SES is linked to a higher rate of cesarean Sect. [8]. Furthermore, we observed more assisted deliveries for women living in non-deprived neighborhoods. This finding has, to our knowledge, not previously been observed. The higher risk of assisted deliveries is caused by the complexity of the pregnancy (and delivery), but it can also partly be a result of the line of care (primary vs. secondary care) [51, 52]. The line of care is associated with mode of delivery: women with low risk pregnancies who received primary care (midwifery-led) during delivery had lower odds of an assisted delivery or cesarean section than women with low risk pregnancies who received secondary care (obstetric-led) [52]. Thus, deliveries that started in secondary care are considered high-risk pregnancies which increases the risk of more cesarean sections. But also the tier of care could partly influence these higher odds of interventions in obstetric-led care. We could not distinguish between planned and emergency cesarean sections as this information was not provided in the claim data. This would have been interesting since emergency cesarean sections have higher rates of maternal and neonatal complications, leading to more treatment in secondary care and higher maternity care costs [53]. 

The total costs for maternity care were significantly higher for women in deprived neighborhoods (156 euros higher, 95%CI 104–208), likely due to a combination of the 23% surcharge for primary care for women in deprived neighborhoods, higher prevalence of hospital delivery (assisted by the primary care midwife) and higher secondary care costs. The costs for primary care were lower in deprived neighborhoods (Table 1), presumably due to their earlier referral to secondary care. The primary care they did receive, however, was more expensive due to the surcharge. Women in deprived neighborhoods more often opted to deliver at the hospital, resulting in additional costs associated with the rental of the delivery room. Higher secondary care costs may reflect the earlier referral to secondary care and the more complex care given in secondary care. Women in deprived neighborhoods received secondary care earlier in their pregnancy, presumably because these pregnancies are more often high-risk pregnancies [8, 20–30]. Secondary care and interventions are more expensive than care and interventions provided by a primary care midwife. Furthermore, interventions in secondary care, such as cesarean section, increase the risk of (additional) complications and the need for corresponding treatments, which may result in a rapid escalation of costs for these women [53]. 

Interestingly, the costs for postnatal maternity help were much lower for women in deprived neighborhoods. As the price per hour for postnatal maternity help has a set maximum rate (and is therefore presumably equal for all health insurers), the lower costs indicate a lower uptake of postnatal maternity help hours in deprived neighborhoods. There could be several explanations for a lower uptake. First, women who use postnatal maternity help have to pay an out-of-pocket fee per hour. This payment was about 4 euros per hour in 2018 [54]. Women with lower SES have less money to spend, and might therefore, choose to use fewer or no hours of postnatal maternity help [55, 56]. This effect was also visible in our analysis. Second, women in deprived neighborhoods are more likely to have a migration background [38]. This could mean that they are less familiar with postnatal maternity help as a possibility, as this is an unique Dutch service, or cultural differences might shape their preference for postnatal maternity help. For example, postnatal support can be provided by family members, or they do not want a stranger in their house for multiple hours each day [57]. Finally, when mother or child was hospitalized during delivery and/or in the first days after the delivery, assistance during delivery and postnatal care was provided in the hospital and not using postnatal maternity help services, leading to lower postnatal maternity help costs. As more women in deprived neighborhoods receive care at the hospital, they might be eligible for fewer hours of postnatal maternity help at home.

In the present study, very and extreme preterm delivery (delivery before 32 weeks as compared to delivery from 32 weeks) was not linked to neighborhood deprivation. Previous studies observed a higher prevalence of preterm deliveries (< 37 weeks) in deprived neighborhoods [20–29]. We were able to assess very and extreme preterm delivery (earlier than 32 weeks of gestation) whereas other studies typically examined preterm delivery (before 37 weeks of gestation). This could suggest that neighborhood deprivation is only associated with preterm delivery (between 32 and 37 weeks of gestation) and not with very preterm (26–32 weeks) or extreme preterm (< 26 weeks) delivery. Bonet et al., however, did find a correlation between neighborhood deprivation and very preterm delivery in France and the United Kingdom [58]. Our sensitivity analysis, in which a less stringent classification of neighborhood classification was used, did reveal a significant difference in very preterm and extreme preterm deliveries between women living in deprived neighborhoods and those in non-deprived neighborhoods. However, we could not find any biomedical explanation for this result. Moreover, due to the low prevalence of very preterm and extreme preterm deliveries in our study and in general, these findings could be incidental. Our estimation of very and extreme preterm delivery relies on data reported voluntarily by hospitals (the DIS data), making this variable less complete than other variables included in our study [31]. Notably, 0.6% of secondary care delivery claims did not include a diagnosis (e.g. very preterm delivery). It is also possible that some of the diagnose codes were omitted when multiple diagnoses were made. We have no insight into how often this was the case. The relative incompleteness may also explain the lower prevalence of very and extreme preterm delivery in our sample compared to other yearly prevalence reported elsewhere. These prevalences are based on registration by all maternity care providers of the Netherlands (between 26 and 32 weeks 1.2% in 2018 and 1.2% in 2019; and before 26 weeks 0.6% and 0.5% respectively) [2]. However, we found no indication that completeness of the preterm deliveries registration differed between deprived and non-deprived neighborhoods.

### Strengths and limitations

A major strength of this study is that we had access to a population-wide dataset. This means that all findings reflect the Dutch population and their outcomes and costs for maternity care. We had access to a large sample, which increased external validity and accuracy of the results and allowed for adjustment for case-mix variables. As the dataset contained information on year of birth, postal code, and previous healthcare costs, it was possible to adjust for these case-mix factors to estimate a more accurate association between neighborhood deprivation and the outcome variables. Invoices from all maternity care services were used in this study, which provided an overview of the provided care over the whole pregnancy and postpartum trajectory (up to six weeks after delivery). Furthermore, this study uses advanced statistical methods to calculate associations between the determinant and outcomes.

This study also had some limitations. The present study did not show which factors of deprived neighborhoods could be responsible for the found association. Exposure to adverse living circumstances increases the likelihood of adverse maternity care outcomes [59]. The present study assessed the association of neighborhood deprivation during pregnancy with adverse outcomes. Not only the neighborhood during pregnancy but also the neighborhood before pregnancy is likely to be crucial for adverse outcomes later in life [59]. We were not able to distinguish which factors contributed to the found effects. For example, we had limited access to demographic and health characteristics of the women. Factors such as parity, lifestyle, and medical or family history were not available in the present study based on nationwide claim data. Parity is often higher in women with lower SES, as well as a higher prevalence of poor lifestyles [60]. Both affect the odds of adverse pregnancy outcomes [61, 62]. However, we did include the total healthcare costs in the previous year (year 2017) as a proxy of women’s medical history. Furthermore, there may be additional reasons why women in deprived neighborhoods are more often treated in secondary care, such as health care preferences. A study from Bolten et al. found that women with planned home birth were more likely to deliver spontaneously and had fewer medical interventions [63]. The present study showed that women living in deprived neighborhoods were more likely to deliver at the hospital than at home, even when the delivery was under supervision of the primary care midwife. This could have a cumulative impact on the intervention rates during delivery of women in deprived neighborhoods, who are more likely to start delivery in secondary care due to higher risk of complications and even in primary care more often have a planned hospital birth.

Second, the dataset included pregnancy trajectories of pregnancies that ended in a delivery. Miscarriages and medical abortions (< 16 weeks gestation) were not included in the analysis, leaving out information on maternity care outcomes and costs. However, miscarriage and medical abortion shorten the pregnancy trajectory, with potentially lower maternity care costs. The prevalence of miscarriages and medical abortions may differ between the types of neighborhoods and could confound the cost differences between the two groups [17]. Therefore, the results of the present study are only generalizable to women with pregnancies that did not end in a miscarriage or abortion.

## Conclusion

The present study showed that women living in deprived neighborhoods in the Netherlands receive secondary care earlier in their pregnancy, have a cesarean section more often and have higher maternity care costs than those in non-deprived neighborhoods. These findings support the needs for increased attention to for socio-economic factors in maternity care in the Netherlands. Both individual SES and living environment have the potential to impact maternity care outcomes and costs. Future studies should assess the distinct impacts of a deprived neighborhood and a low socioeconomic status on maternity care outcomes and costs. We encourage policy advisors to consider interventions targeting both the individual SES and living environment to improve maternity care outcomes and decrease maternity care costs.

### Electronic supplementary material

Below is the link to the electronic supplementary material.

Supplementary Material 1

## Data Availability

The data that support the findings of this study are available from Vektis but restrictions apply to the availability of these data, which were used under license for the current study, and so are not publicly available. Data are however available from the corresponding authors upon reasonable request and with permission of Vektis.

## Footnotes

1 Fetal mortality rate: the number of fetal deaths at or after 22 weeks gestation until and excluding birth per 1000 births.

2 Apgar score: method to evaluate condition of a newborn infant. A low score reflects poor condition.

## Abbreviations

SES: Socio-economic status
NZa: Nederlandse Zorgautoriteit (Dutch Healthcare Authority)
DBC: Diagnose Behandel combinatie (Diagnosis Treatment Combination)
IGO: Integrated maternity care organization
DIS: DBC information system
SD: Standard deviation
IQR: Inter-quartile range
METC: Medical Ethics Assessment Committee
OR: Odds ratio
