# Supplementary material for: Do women living in a deprived neighborhood have higher maternity care costs and worse pregnancy outcomes? A retrospective population-based study
Source: BMC Health Serv Res. 2024 Mar 20;24:360. doi: 10.1186/s12913-024-10737-2 (PMC10956252; doi:10.1186/s12913-024-10737-2)
Supplement: Supplementary file 1 — Supplementary Material 1 [file 12913_2024_10737_MOESM1_ESM.pdf]

Supplementary information

TABLE S1.

Model S1 Multinomial regression moment of referral, no referral as reference category, deprived when she lived in a deprived neighborhood for one or more quarters

	Referral at delivery or postpartum				Referral during pregnancy				Start in secondary care			
	Crude model		Adjusted model		Crude model		Adjusted model		Crude model		Adjusted model	
	OR (95%CI)	P-value	OR (95%CI)	P-value	OR (95%CI)		OR (95%CI)	P-value			OR (95%CI)	P-value
Deprived neighborhood	1.25 (1.19 – 1.31)	<0.001*	1.07 (1.01 – 1.12)	0.02*	1.59 (1.52 – 1.66)	<0.001*	1.55 (1.48 – 1.64)	<0.001*	1.52 (1.43 – 1.62)	<0.001*	1.52 (1.42 – 1.63)	<0.001*
Age												
Younger than 25 years			Ref				Ref				Ref	
25-30 years			0.78 (0.74 – 0.82)	<0.001*			0.85 (0.80 – 0.90)	<0.001*			0.99 (0.92 – 1.08)	0.94
30-35 years			0.61 (0.57 – 0.64)	<0.001*			0.79 (0.74 – 0.83)	<0.001*			1.00 (0.92 – 1.09)	0.99
35 years or older			0.54 (0.51 – 0.58)	<0.001*			1.00 (0.95 – 1.06)	0.91			1.59(1.46 – 1.73)	<0.001*
Healthcare costs 2017												
< €123			Ref				Ref				Ref	
€123 – €254			1.04 (0.99 – 1.09)	0.09			1.14 (1.09 – 1.20)	<0.001*			0.94 (0.86 – 1.01)	0.11
€254 – €612			1.20 (1.14 – 1.26)	<0.001*			1.44 (1.38 – 1.52)	<0.001*			1.43 (1.32 – 1.54)	<0.001*
€612 – €1742			1.23 (1.17 – 1.29)	<0.001*			1.66 (1.58 – 1.75)	<0.001*			2.27 (2.11 – 2.44)	<0.001*
€1742 – €4911			0.72 (0.69 – 0.76)	<0.001*			1.04 (0.99– 1.09)	0.08			1.74 (1.62 – 1.87)	<0.001*
> €4911			0.80 (0.75 – 0.84)	<0.001*			1.79 (1.70 – 1.88)	<0.001*			3.31 (3.09 – 3.55)	<0.001*
Population density												
Very strongly urbanized			1.15 (1.10 – 1.20)	<0.001*			0.93 (0.89 – 0.97)	<0.01*			0.79 (0.75 – 0.84)	<0.001*
Strongly urbanized			1.07 (1.03 – 1.12)	<0.01*			1.03 (0.99 – 1.08)	0.13			0.99 (0.94 – 1.05)	0.78
Moderately urbanized			Ref				Ref				Ref	
Hardly urbanized			0.95 (0.91 – 0.99)	0.04*			0.97 (0.92 – 1.01)	0.14			0.80 (0.75 – 0.85)	<0.001*
Not urbanized			0.84 (0.80 – 0.89)	<0.001*			0.94 (0.90 – 0.99)	0.01*			0.78 (0.73 – 0.84)	<0.001*

Note.

* P-value lower than alpha of 0.05.

TABLE S2.

Model S2 Multinomial regression mode of delivery, adjusted for case-mix factors, deprived when she lived in a deprived neighborhood for one or more quarters

	Assisted delivery				Cesarean section			
	Crude model OR (95%CI)	P-value	Adjusted model OR (95%CI)	P-value	Crude model OR (95%CI)	P-value	Adjusted model OR (95%CI)	P-value
Deprived neighborhood	0.85 (0.79 – 0.92)	<0.001*	0.77 (0.71 – 0.84)	<0.001*	1.17 (1.12 – 1.22)	<0.001*	1.21 (1.15 – 1.27)	<0.001*
Age								
Younger than 25 years			Ref				Ref	
25-30 years			1.11 (1.02 – 1.21)	0.02*			1.28 (1.20 – 1.37)	<0.001*
30-35 years			0.91 (0.84 – 0.99)	0.03*			1.51 (1.42 – 1.61)	<0.001*
35 years or older			0.78 (0.71 – 0.85)	<0.001*			2.07 (1.94 – 2.20)	<0.001*
Healthcare costs 2017								
< €123			Ref				Ref	
€123 – €254			0.93 (0.86 – 0.99)	0.04*			1.13 (1.07 – 1.19)	<0.001*
€254 – €612			0.98 (0.91 – 1.05)	0.53			1.24 (1.18 – 1.31)	<0.001*
€612 – €1742			0.95 (0.89 – 1.03)	0.21			1.28 (1.23 – 1.37)	<0.001*
€1742 – €4911			0.65 (0.60 – 0.70)	<0.001*			1.04 (0.98 – 1.10)	0.18
> €4911			0.56 (0.51 – 0.61)	<0.001*			1.42 (1.35 – 1.50)	<0.001*
Population density								
Very strongly urbanized			1.23 (1.14 – 1.31)	<0.001*			1.04 (0.99 – 1.09)	0.08
Strongly urbanized			1.13 (1.06 – 1.22)	<0.001*			1.06 (1.02 – 1.11)	<0.01*
Moderately urbanized			Ref				Ref	
Hardly urbanized			1.00 (0.93 – 1.08)	0.96			1.00 (0.95 – 1.05)	0.99
Not urbanized			0.99 (0.92 – 1.08)	0.99			1.02 (0.97 – 1.08)	0.41

Note.

* P-value lower than alpha of 0.05.

TABLE S3.

Model S3 Ordinal regression preterm delivery, deprived when she lived in a deprived neighborhood for one or more quarters

	Crude model		Adjusted model	
	OR (95%CI)	P-value	OR (95%CI)	P-value
Deprived neighborhood	1.32 (1.07 – 1.61)	0.007*	1.30 (1.02 – 1.64)	0.03*
Age				
Younger than 25 years			Ref	
25-30 years			0.96 (0.72 – 1.29	0.78
30-35 years			0.96 (0.73 – 1.29)	0.80
35 years or older			0.97 (0.72 – 1.32)	0.83
Healthcare costs 2017				
< €123			Ref	
€123 – €254			1.20 (0.89 – 1.63)	0.23
€254 – €612			1.50 (1.12 – 2.00)	0.006*
€612 – €1742			1.47 (1.10 – 1.96)	0.01*
€1742 – €4911			1.49 (1.12 – 1.99)	0.006*
> €4911			1.88 (1.43 – 2.50)	<0.001*
Population density				
Very strongly urbanized			1.24 (0.98 – 1.59)	0.08
Strongly urbanized			1.10 (0.86 – 1.41)	0.45
Moderately urbanized			Ref	
Hardly urbanized			1.04 (0.79 – 1.36)	0.80
Not urbanized			1.31 (1.00 – 1.72)	0.05*

Note.

Preterm delivery was classified in three categories: (close to) term delivery (>32 weeks, reference category), preterm delivery (26-32 weeks gestation), extreme preterm delivery (<26 weeks gestation).

* P-value lower than alpha of 0.05

TABLE 4.
Model S4 Linear regression total maternity care costs, in euros, deprived when she lived in a deprived neighborhood for one or more quarters

	Crude model<br>Coefficients (95%CI)	P-value	Adjusted model<br>Coefficients (95%CI)	P-value
Deprived neighborhood	206 (165 – 246)	<0.001*	194 (148 – 241)	<0.001*
Year of delivery 2019	150 (121 – 179)	<0.001*	146 (116 – 175)	<0.001*
Age				
Younger than 25 years			Ref	
25-30 years			34 (-19 – 87)	0.21
30-35 years			19 (-32 – 71)	0.46
35 years or older			244 (189 – 300)	<0.001*
Healthcare costs 2017				
< €123			Ref	
€123 – €254			174 (127 – 221)	<0.001*
€254 – €612			376 (329 – 423)	<0.001*
€612 – €1742			572 (525 – 619)	<0.001*
€1742 – €4911			309 (262 – 356)	<0.001*
> €4911			730 (683 – 778)	<0.001*
Population density				
Very strongly urbanized			-34 (-79 – 8)	0.11
Strongly urbanized			9 (-33 – 50)	0.69
Moderately urbanized			Ref	
Hardly urbanized			-5 (-51 – 40)	0.81
Not urbanized			-14 (-62 – 34)	0.56
Intercept	6793 (6767 – 6819)	<0.001*	6398 (6331 – 6465)	<0.001*

Note

* P-value lower than alpha of 0.05
